# Books and Authors

**Published:** 1906-06

**Authors:** 


### BOOKS AND AUTHORS.

**Pharmacology and Therapeutics.** By **Reynold Webb Wilcox,** M.A., M.D., LL.D., Professor of Medicine at the New York Post-Graduate Medical School and Hospital, and Attending Physician to the Hospital; Consulting Physician to the Nassau Hospital, etc. Small octavo pp. 1010. Sixth edition. Philadelphia: P. Blakiston's Son & Co. 1905. (Price, Cloth $3.00 net).

This work is based on the fifth edition of White and Wilcox's Materia Medica and Therapeutics. In order to bring that work into harmony with the United States Pharmacopeia, and, further, because so much additional material has been introduced into the five previous editions, it became necessary to rewrite the book. The eighth decennial revision of the pharmacopeia furnished the opportunity, hence this is practically a new book and has been given a new title; or, to speak more exactly, the author has divided the subject into two works or separate parts,—one being materia medica and pharmacy, and the other pharmacology and therapeutics. The first was noticed in the JOURNAL for March, p. 519, and now we are pleased to present the latter to the attention of our readers.

In detailing the pharmacologic and therapeutic actions of drugs, the author is a master. He makes fifteen divisions of the subject, dealing with their actions on almost every tissue and organ of the body,—on organisms which infect the human body, drugs acting on the blood, the cardiac mechanism, the vessels, the skin, the urinary system, the bodily heat, the respiration, the digestive apparatus, the nervous and muscular systems, the organs of generation, antitoxins and serums, organic extracts, drugs acting on metabolism and drugs which have no marked therapeutic properties.

It will thus be observed that the entire field is covered and, we may add with great propriety, most admirably. The student of pharmacology and therapeutics, whether undergraduate or practitioner, can ill afford to do without this book.

---

**Nervous and Mental Diseases.** By **Archibald Church,** M.D., Professor of Nervous and Mental Diseases and Medical Jurisprudence in Northwestern University Medical School, Chicago; and **Frederick Peterson,** M.D., President of the State Commission in Lunacy, New York; Clinical Professor of Neurology and Psychiatry, Columbia University. Fifth edition, revised and enlarged. Octavo volume of 937 pages, with 341 illustrations. Philadelphia and London: W. B. Saunders & Co., 1905. (Cloth, $5.00, Half Morocco, $6.00 net).

If the physician of fifty years ago could look upon this treatise he would be amazed that such a vast volume were needed to adequately describe the diseases of the nerves and mind. The textbook on the theory and practice of medicine of those days was not larger and the surgical works of Ferguson, Druitt, and Liston were smaller. That five editions of this treatise should be required to supply the demand that it has created for itself since 1899, bespeaks an estimate of its worth that ought to be very gratifying to the authors. The improvements of the section on nervous diseases consist chiefly in adding many short sentences or interpolations which advances have made necessary, and which serve to freshen the text and keep it abreast with the latest thought on the subject. Some new engravings, too, have replaced those that were considered unnecessary or obsolete.

Several changes, likewise, have been made in the second part, that dealing with insanity. Dr. Peterson has added the classification of Kraepelin, and two new chapters,—one upon manic-depressive insanity, and one on dementia precox,—have been written. These several changes serve to keep the work apace with the progression of psychiatry that is ever occuring. In its present condition the treatise represents the latest and best literature of the subjects with which it deals and is an ornament to the science of medicine.

---

**The Medical Directory of New York, New Jersey and Connecticut.** Published by the New York State Medical Association, 64 Madison Avenue, New York. Volume VII. Committee of Publication: **Charles Ellery Denison,** chairman; **E. Eliot Harris, Thomas F. Reilly, Henry Roth, and William Ridgely Stone.** New York: The Trow Printing and Binding Company, 1905.

The seventh volume of this valuable directory is built upon the same lines as its immediate predecessors. The aim has been to publish a complete list of all legally registered physicians,—a most difficult yet desirable task to accomplish. It is to be remarked, however, that it has been done with a very close approach to correctness in this edition. The total number of names recorded is 15,472, of which 11,923 reside in the state of New York, 2,253 in New Jersey, and 1,296 in Connecticut. Of these, 5,852 are registered in Manhattan and Bronx; 1,509 in Brooklyn; 130 in Queens, and 62 in Richmond, making a total of 7,553 in Greater New York, leaving 4,370 for the remainder of the state. Those not receiving the directory as members can obtain it on remitting $2.50 to 64 Madison Avenue, New York.

---

**A Textbook of Modern Materia Medica and Therapeutics.** By **A. A. Stevens,** A.M., M.D., Lecturer on Physical Diagnosis, University of Pennsylvania; Professor of Pathology, Woman's Medical College of Philadelphia. Fourth edition, revised. Octavo of 670 pages. Philadelphia and London: W. B. Saunders & Company, 1905. (Price, cloth, $3.50 net.)

Like most others who have written textbooks on materia medica and therapeutics, this author has found it essential to prepare a new edition in order to make his work conform to the recent revision of the pharmacopeia. Stevens has long been known as a pains-taking teacher and writer, and he has made this book an exponent of the best thought and latest knowledge relating to the topics embraced in the title. While it is adapted to the special needs of the student of medicine, it is also an excellent reference book for the practitioner. Its arrangement is excellent and its material is clear, concise and, withal, adequate to the every day needs of both undergraduate and practising physician. Some of its features are distinctive and the work as a whole compares favorably with any similar treatise on the medical book market.

---

**Food and Diet in Health and Disease**. A Manual for Practitioners of Medicine, Students, Nurses and the Lay Reader. By **Robert F. Williams,** M.A., M.D., Professor of Principles and Practice of Medicine in the Medical College of Virginia, Richmond. Duodecimo volume of 392 pages. Lea Brothers & Co., Publishers, Philadelphia and New York, 1906. (Cloth, net, $2.00).

The author of this excellent monograph dedicates it to his longtime friend, Professor George Ben Johnston, of Richmond, who may well feel a pride in having so good a book inscribed to his affectionate regard. Dr. Williams is a master of his subject and has presented it in a most entertaining as well as in an instructive manner.

The book is divided for convenience, into two parts: part I, dealing with food in health, and part II, with food in disease. In part I. the needs of the body for different kinds of foods and the manner in which they are utilised are explained. The principles of cooking foods and detailed descriptions of the different articles of food in common use are given, with chapters on the proper nutriment of infants, children, adults, and the aged.

Part II. deals with the variations from the normal diet in health, necessitated by the more common diseases, and includes a chapter on general methods to be observed in feeding the sick, as well as the special directions for nourishment in diseases of different kinds.

The facts are presented with a commendable simplicity as well as a clearness that makes it easy to comprehend and remember them. It is just such a book as should claim the attention of junior physicians who are sometimes neglectful of the principles which it inculcates.

---

**The Practice of Medicine**. A textbook for practitioners and students, with special reference to diagnosis and treatment. By **James Tyson,** M.D., Professor of Medicine in the University of Pennsylvania, and physician to the Hospital of the University; physician to the Pennsylvania Hospital etc. Fourth edition, revised and enlarged, with 240 illustrations, including colored plates. Octavo, pp. 1350. Philadelphia: P. Blakiston's Son & Company, 1012 Walnut St. 1906. (Price, cloth, $5.50).

It is a genuine pleasure to see a new edition of this excellent textbook which stands in the fore-front of works on practice. It is but two years since the last previous edition was published, but the demands for a new one were urgent, and author and publisher have each met them most satisfactorily. Tyson has been known for a long time as one of the best teachers of practice in the country, and he writes with a clearness and precision that makes his book attractive as well as instructive.

A careful revision has been made of this edition and over sixty pages of new material have been incorporated in the text. In the section on animal parasites important changes have been made, and the whole subject has been carefully revised by Dr. Allen J. Smith, the author's colleague, who is everywhere recognised as authority on this most important topic. Numerous illustrations have also been introduced in this section, thus making one of the most complete expositions of this very practical subject.

Many other changes have been made throughout the volume with a view to make it a modern working textbook containing everything of importance to student and practitioner, and bringing it forward to the immediate present in all its essential data and teachings.

---

**Culbreth's Materia Medica.** A Manual of Materia Medica and Pharmacology for Students and Practitioners of Medicine and Pharmacy. Comprising all Organic and Inorganic Drugs which are and have been official in the United States Pharmacopeia, together with important Allied Species and Useful Synthetics. By **David M. R. Culbreth,** Ph.G., M.D., Professor of Botany, Materia Medica and Pharmacology in the University of Maryland, Departments of Medicine, Pharmacy and Dentistry. Fourth edition. Revised to accord with the new U. S. Pharmacopeia, 8th Decennial Revision. Octavo, 976 pages, 487 illustrations. Lea Brothers & Co., Publishers, Philadelphia and New York. 1906. (Cloth, $4.75, net).

In nine years three editions of this book were exhausted, and the author has allowed some time to pass since the last previous edition was sold completely out, in order to prepare this one with unusual care, that it may represent the last thought and reflect the very latest knowledge relating to materia medica and pharmacy. Culbreth has had uncommon opportunities to become familiar with the practical side of his topics, having been in the retail drug business for twenty years. Then he became a teacher; hence it may be said that the work before us is the result of both shop and laboratory experience, carefully garnered and served up to students and practitioners in a most scientific as well as practical manner. The revision of this edition has been so extensive as to make it almost a new work. New illustrations have been made to supplant old ones, and the entire treatise has an air of freshness that should commend it to a large audience of readers.

---

**A Textbook of Pharmacology and Therapeutics,** or The Action of Drugs in Health and Disease. By **Arthur R. Cushny,** M.A., M.D., Aberd., Professor of Pharmacology in the University College, London; formerly Professor of Materia Medica and Therapeutics in the University of Michigan. In one octavo volume of 752 pages, with 52 illustrations. Lea Brothers & Co., Publishers, Philadelphia and New York. 1906. (Cloth $3.75 net).

It is not very long ago that we had the pleasure of commending the third edition of this textbook to our readers, and it is but seven years since the first edition appeared. This frequent demand for new editions supports our judgment first expressed regarding the value of the treatise. Since the book was first published Cushny has been called to the University College, London, where he holds the professorship of pharmacology. Nevertheless, he has not forgotten the claims of his American readers, and has prepared this edition to conform to the recent revision of the United States Pharmacopeia. Many changes have been made in the text and the work may be said to reflect the latest information that is in possession of the most advanced of teachers. Cushny may be credited, with great justice, as having presented to the student and practitioner, one of the very best working textbooks on the topics of which it treats, and of having written it in an attractive style as to material and phraseology.

---

**Progressive Medicine, Vol. VIII, No. I, March, 1906.** A quarterly Digest of Advances, Discoveries and Improvements in the Medical and Surgical Sciences. Edited by **Hobart Amory Hare,** M.D., Professor of Therapeutics and Materia Medica in the Jefferson Medical College of Philadelphia. Octavo, 304 pages, with 7 engravings. Lea Brothers & Co., Publishers, Philadelphia and New York. (Per annum in four cloth-bound volumes, $9.00; in paper binding, $6.00, carriage paid to any address).

The spring number of this useful digest contains abstracts of the surgery of the head, neck, and thorax, by Charles H. Frazier; of infectious diseases, including acute rheumatism, croupous pneumonia, and influenza, by Robert B. Preble; of the diseases of children, by Floyd M. Crandall; of rhinology and laryngology, by D. Braden Kyle; and of otology, by B. Alexander Randall. The larger part of the book,—nearly one-half,—is taken up with the surgery of the head, neck, and thorax, which is an exceedingly well presented digest of the recent literature of the subjects. The entire volume contains the material most needed by the busy physician, who must obtain information quickly relating to some important case in hand.

---

**Man and His Poisons.** A practical Exposition of the Causes, Symptoms and Treatment of Self-Poisoning. By **Albert Abrams,** A.M., M.D., Consulting Physician to the Denver National Hospital for Consumptives, the Mount Zion, and the French Hospitals, San Francisco. Illustrated, pp. 268. New York: E. B. Treat & Company. 1906. (Price, $1.50 net).

This book in reality is a series of essays in which the author displays considerable learning and some practical knowledge of the causes, pathology, and treatment of autointoxication. His remarks on the psychology of eating contain much of value. What to eat and how to eat are problems that may well engage the attention of every physician who would deal successfully with the diseases of the alimentary tract. The volume will be found of interest to many individuals who are not physicians, and deserves a wide circulation.

